# Highly Sensitive and Stable Copper-Based SERS Chips Prepared by a Chemical Reduction Method

**DOI:** 10.3390/nano11102770

**Published:** 2021-10-19

**Authors:** Pei Dai, Haochen Li, Xianzhi Huang, Nan Wang, Lihua Zhu

**Affiliations:** School of Chemistry and Chemical Engineering, Huazhong University of Science and Technology, Wuhan 430074, China; peidai94@hust.edu.cn (P.D.); u201810449@hust.edu.cn (H.L.); huangxzh26@mail2.sysu.edu.cn (X.H.); nwang@hust.edu.cn (N.W.)

**Keywords:** surface-enhanced Raman scattering, copper, SERS chip, chemical reduction, hydrophobicity

## Abstract

Cu chips are cheaper than Ag and Au chips for practical SERS applications. However, copper substrates generally have weak SERS enhancement effects and poor stability. In the present work, Cu-based SERS chips with high sensitivity and stability were developed by a chemical reduction method. In the preparation process, Cu NPs were densely deposited onto fabric supports. The as-prepared Cu-coated fabric was hydrophobic with fairly good SERS performance. The Cu-coated fabric was able to be used as a SERS chip to detect crystal violet, and it exhibited an enhancement factor of 2.0 × 106 and gave a limit of detection (LOD) as low as 10–8 M. The hydrophobicity of the Cu membrane on the fabric is favorable to cleaning background interference signals and promoting the stability of Cu NPs to environment oxidation. However, this Cu SERS chip was still poor in its long-term stability. The SERS intensity on the chip was decreased to 18% of the original one after it was stored in air for 60 days. A simple introduction of Ag onto the clean Cu surface was achieved by a replacement reaction to further enhance the SERS performances of the Cu chips. The Ag-modified Cu chips showed an increase of the enhancement factor to 7.6 × 106 due to the plasmonic coupling between Cu and Ag in nanoscale, and decreased the LOD of CV to 10–11 M by three orders of magnitude. Owing to the additional protection of Ag shell, the SERS intensity of the Cu-Ag chip after a two-month storing maintained 80% of the original intensity. The Cu-Ag SERS chips were also applied to detect other organics, and showing wide linearity range and low LOD values for the quantitative detection.

## 1. Introduction

Surface-enhanced Raman spectroscopy (SERS), as a highly sensitive vibrational spectroscopy [1], is one of the most commonly used on-field spectroscopic detection techniques with advantages of convenience, rapidness and high sensitivity [2]. Typical SERS active substrates are noble metals (Au, Ag, Cu) at nanoscale. A laser can excite their localized surface plasmons resonances (LSPR) and amplify electromagnetic fields, finally leading to the SERS with electromagnetic enhancement mechanism (EM) [3,4]. Among these noble metals, Ag-based substrates produce the strongest Raman enhancement and Au-based substrates have the best long-term stability in air, whereas Cu-based substrates attract the least attention because of their insignificant SERS enhancement and poor stability, although Cu is much cheaper. 

Due to the poorer LSPR of Cu, its electromagnetic enhancement is weaker than that of Ag and Au. The SERS enhancement factor obtained for simple copper substrates were reported to be 10^3^–10^7^ [5,6], being lower than that of silver (10^6^–10^14^) and gold (10^4^–10^9^) [7,8]. In order to improve the SERS performances of Cu substrates, two strategies are often employed. One is to construct the SERS substrates with compact arrangement of nanoparticles. Kowalska et al. used high pressure for the decomposition of copper hydride (CuH) to prepare SERS platforms with uniformly distributed copper nanocrystals; the obtained substrates showed good SERS performance with enhancement factor up to 10^6^–10^7^ [9]. Rao et al. assembled spherical nanocopper into three-dimensional nanoporous Cu leaves by modifying Cu NPs with isooctane and polyethylene glycol, and found that the porous 3D structure gave an enhancement factor of 1.2 × 10^6^ [10]. The other is to combine copper with other materials [11]. Dizajghorbani-Aghdam et al. co-deposited Cu NPs with a graphitic carbon nitride (gCN) support, and found that the Cu/gCN hybrids showed strong absorption in the visible light to near-IR range, resulting in an enhancement factor of 10^7^ [12]. 

Copper is chemically active in air, and hence Cu NPs are easy to aggregate irregularly and to be oxidized, leading to the decrease of the enhancement effect. Loading Cu NPs onto the fixed substrates, such as Si wafer [13,14], glass [15], polystyrene spheres [16] and graphene oxide (GO) [17], could prevent the random aggregation of colloidal copper. A compact arrangement of Cu NPs may produce abundant hot spots with fairly high enhancement effect with improved SERS performances. Surface coating of noble metal nanoparticles is a common method to prevent oxidation [18,19]. The size and morphology of nanocopper can be controlled by modifying surfactants on the surface of copper, and the obtained coating can also hamper the aggregation and oxidation of Cu NPs. Zhang et al. encapsulated Cu NPs in a graphene shell (thickness: 1 nm) to improve the stability of Cu NPs [20]. Compared with traditional SERS coatings of metallic NPs, such as SiO_2_ [21], polymers [22] and amorphous carbon [23], the few layers of graphene could strengthen the plasmonic coupling between graphene and Cu [24], exhibiting an enhancement factor of 1.15 × 10^6^.

Copper-based SERS chips have been prepared with various methods though chemical reduction [25,26], electrochemical deposition [27], aerosol direct writing [28], subsequent dealloying process [29], magnetron sputtering [30], laser ablation [12] and chemical vapor deposition (CVD) [20]. Among these methods, the chemical and electrochemical reduction processes are ubiquitous and convenient to operate without the needs of high temperature, high level of vacuum and expensive instruments. However, the surface of Cu NPs produced by solution chemistry techniques may be contaminated by the added stabilizing agent, such as polyvinyl pyrrolidone [31], sodium dodecylbenzene sulphonate [32] or cetyltrimethylammonium bromide [33], the residues of which easily lead to disturbances in high-sensitivity detection.

In the present work, we successfully developed the Ag-modified Cu SERS chip with high sensitivity and stability by a chemical reduction method. By loading Cu NPs on the fabric, the random aggregation of colloidal copper was avoided. The compact arrangement of Cu NPs leads to the hydrophobicity and sensitivity of the copper-based SERS chip. Without any modifier to hinder SERS performance and create interference signals, the Cu substrate had a clean background. Silver could be directly deposited onto the surface-clean Cu-coated fabric by a replacement reaction. The introduction of Ag further strengthened the plasmonic coupling between Cu and Ag, therefore contributing to an improved sensitivity. The hydrophobicity of the copper membrane was of great importance because a hydrophobic surface could improve the stability of materials [34]. Being benefit from the hydrophobic effect of the hydrophobic surface and the protection from the thin silver shell, the as-prepared SERS chips had good stability in air. We should note that it looked strange but is very interesting that the SERS performances of the mildly oxidized SERS substrate could be partially recovered by a vacuum deoxygenation treatment. Owing to the contribution of chemical enhancement mechanism (CM) in this Cu-Ag chip, the target molecules with required energy levels could be selective enhanced and quantitative detected through the photo-induced charge transfer (PICT).

## 2. Experimental Section

### 2.1. Reagents

N_2_H_4_·H_2_O (85%), H_2_O_2_ (30%), crystal violet (CV) and ethanol were purchased from Sinopharm Chemical Reagent Co., Ltd (Shanghai, China). Cu(CH_3_COO)_2_·H_2_O and AgNO_3_ were purchased from Shanghai Lingfeng Chemical Reagents Co., Ltd (Shanghai, China). Paraquat (PQ) was purchased from Macklin (Shanghai, China). Sibutramine hydrochloride (SH) was obtained from the National Institute for the Control of Pharmaceutical and Biological Products (Beijing, China). All the chemicals were analytically pure and used as received without further purification. Deionized water was used throughout the experiments.

### 2.2. Preparation of Flexible Hydrophobic Ag Modified Copper SERS Chips

The flexible SERS chips were chemically prepared by depositing hydrophobic Cu membrane on fabrics and then coating sliver on the Cu membrane through a replacement reaction. In the first step, copper was coated on fabrics with a modified chemical deposition procedure as reported [35]. Fabrics were cut into pieces with sizes of 2 cm × 2 cm, followed by fully washing with water and ethanol. After being dried, the cleaned fabric was fully immersed in a solution of copper acetate (20 mL, 10 g L^−1^) for several minutes. Then, 800 μL of 80% hydrazine hydrate was added to it drop by drop under mild stirring. The reaction in the mix solution was kept at room temperature for 6 h, which allowed the deposition of copper particles onto the fabric. Afterward, the prepared Cu-coated fabric was taken out and washed with water and ethanol, followed by vacuum drying at 60 °C for 1 h. The obtained Cu membrane on the fabric was confirmed to be hydrophobic. In the present work, this sample was also used as a SERS chip, being referred to as a hydrophobic Cu chip. 

In the second step, Ag particles were further deposited on the above obtained Cu-coated fabric by immersing it in a solution of AgNO_3_ (0.01 M) in ethanol for 1 min. Then, the fabric was washed with water and ethanol, and vacuum dried at 60 °C for 1 h. This product was flexible hydrophobic Ag modified copper SERS chips, being referred to as a hydrophobic Cu-Ag chip. If not specified elsewhere, these two types of the chips were used in the SERS measurements after they were carefully cut into small pieces with a size of 0.5 cm × 0.5 cm.

### 2.3. Characterization

Scanning electron microscopy (SEM) characterization was performed on a GeminiSEM 300 (ZEISS, Heidenheim, Germany). X-ray diffraction (XRD) patterns were recorded by a SmartLab-SE diffractometer (Rigaku, Tokyo, Japan). Contact angles were obtained on an OCA20 optical contact angle meter (Dataphysics, Filderstadt, Germany). UV–vis diffuse reflectance spectra were obtained by a UV-3600 UV-VIS-NIR spectrophotometer (Shimadzu, Kyoto, Japan) in diffuse reflectance mode. XPS analysis was conducted on a K-Alpha X-ray photoelectron spectrometer (Thermo Scientific, Carlsbad, CA, USA).

### 2.4. SERS Detection

The specified SERS chip was immersed in the ethanol solution of analytes for 10 min, and then it was taken out for SERS detection. The SERS detection was performed with a ATR8100 portable Raman spectrometer (Optosky, Xiamen, China). A 785 nm laser was used as an excitation source with a laser power of 50 mW, and the exposure time of 5 s was set without accumulation. During the measurement, the fabric support in the chip was kept wet by adding ethanol. The detection was performed five times at different positions on the tested SERS chip, and the averaged spectrum was used for further analysis.

The Raman enhancement factor was calculated with the following equation:

(1) $$EF=\frac{I_{SERS}\times N_{NR}}{N_{SERS}\times I_{NR}}$$

where *I*_*SERS*_ and *I*_*NR*_ are the intensity of the same peaks position in the SERS spectrum and normal Raman spectrum, respectively, *N_SERS_* represents the number of adsorbed molecules in the SERS analysis and *N_NR_* is the number of molecules in the scattering volume.

### 2.5. Stability Experiment

To evaluate the oxidation resistance of the substrate, a newly made SERS chip was firstly used to detect the original SERS signal of CV (I_0_). Then, it was stored in the air for different periods of time and then measured the SERS intensity (I_t_). The time course of the I_t_/I_0_ value was used to evaluate the stability of the chip for its endurance to the oxidation of substrate.

To recover of the SERS performance of the oxidized SERS substrate, the “oxidized” chip was vacuum dried at 60 °C for 2 h. The vacuum dried “recovered” chip was used for the SERS detection. Here, the so-called “oxidized” chip was obtained by immersing the “freshly prepared” chip for about 30 min in the ethanol solution of 0.01 M H_2_O_2_, which was prepared by dissolving a required amount of H_2_O_2_ (30%) into ethanol. After it was washed with ethanol and air dried, it was used to measure the SERS performance.

## 3. Results and Discussion

### 3.1. Characterizations of the Cu and Cu-Ag Chips

**Figure 1:**
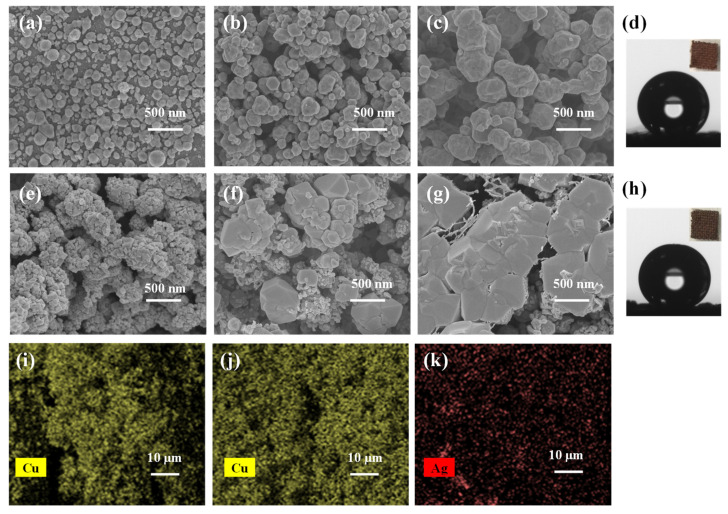
Characterization of Cu and Cu-Ag chips. (**a**–**c**,**e**–**g**) SEM images of the Cu coating with Table 1. h, (**b**) 6 h, (**c**) 12 h and Cu-Ag coating with the replacement time of (**e**) 1 min, (**f**) 3 min and (**g**) 5 min. (**d**,**h**) Contact angles of the coatings of 6 h Cu (**d**) and 6 h Cu-1 min Ag (**h**) on the fabric together with their photos in the inset. (**i**) EDS mapping of Cu on the Cu coating. (**j**,**k**) EDS mapping of Cu (**j**) and Ag (**k**) on the Cu-Ag coating.

In the Cu chip, the fabric was used as the carrier to locate copper nanoparticles and prevent the spontaneous aggregation of them, while Cu NPs were evenly deposited on the fabric by a simple chemical reduction method with hydrazine hydrate as a reducing agent. The as-prepared Cu-coated fabric chip had a deep red color with copper evenly deposited on the filaments. The SEM image of the Cu coating on the fabric (Figure 1a–c) showed that the amount of deposited copper increased and the Cu NPs became bigger with a prolonged deposition time. For the Cu coating with deposition time of 6 h (being referred to as 6 h Cu), the Cu NPs with sizes of about 100–200 nm were packed together closely (Figure 1b). The packing-induced micro-nano structure of the deposited Cu NPs made the surface of the fabric chip hydrophobic, yielding a contact angle of 144.0° (Figure 1d) for 6 h Cu. The EDS mapping of Cu on the Cu coating (Figure 1i) confirmed the even distribution of Cu on the surface of the fabric. 

After the 6 h Cu sample was treated by immersion in a solution of AgNO3 (0.01 M) in ethanol, the Cu chip was converted to the Cu-Ag chip, which was similarly characterized. The SEM image (Figure 1e–g) demonstrated that the Ag NPs covered on Cu NPs generated from the discrete small particles to the monolithic structure. As shown in Figure 1e, the aggregated Cu NPs were covered by much smaller particles on the Cu-Ag coating with 1 min replacement time (6 h Cu-1 min Ag). This is possibly related to the following processes: the replacement reaction led to the generation of Ag NPs with smaller particle sizes and also thinned the out-most Cu particles through the partial dissolution of Cu. Due to the deposition of Ag, the fabric chip became dark in color, indicating the deposition of Ag NPs with small sizes. The EDS mapping of Cu and Ag on the Cu-Ag chip (Figure 1j,k) confirmed the even distribution of both Cu and Ag on the surface of the fabric. The deposition amount of Ag was little, and atomic ratio of Cu: Ag was around 7:1. The easy detection of Cu under the Ag layer by EDS mapping also signed that the outermost layer of Ag was very thin. This thin layer did not change the hydrophobic nature of the pre-obtained Cu layer, and hence the contact angle on the surface of the Cu-Ag chip was still as large as 141.5° (Figure 1h). The relationship between SERS performance and structural morphology of Cu and Cu-Ag chip will be discussed later. 

**Figure 2:**
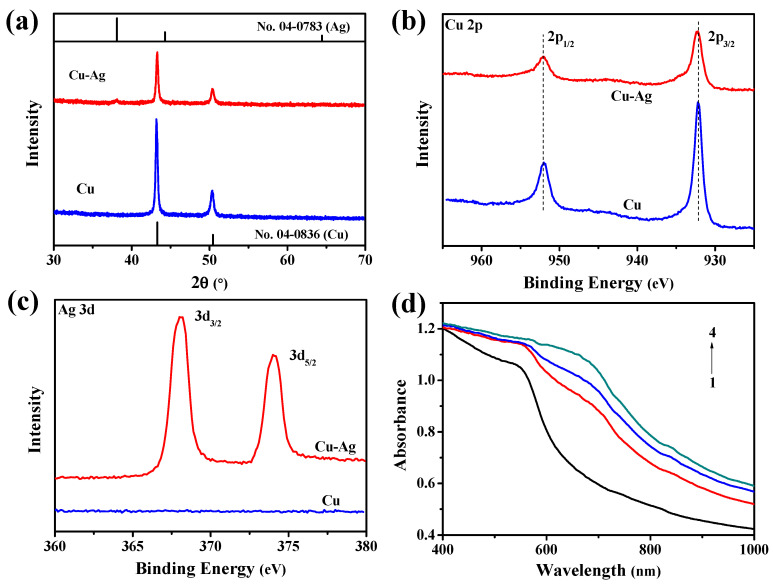
XRD patterns (**a**), XPS high resolution spectra of Cu 2p (**b**), Ag 3d (**c**) of the Cu and Cu-Ag chips. (**d**) VIS-NIR diffuse reflectance spectra of Cu-based fabrics with the Ag replacement Table 1. 0, (2) 1, (3) 3 and (4) 5 min in the preparation.

The crystal structures and chemical structures of the Cu and Cu-Ag chips were investigated by XRD and XPS analysis. As shown in Figure 2a, the XRD patterns of the Cu coating showed two peaks at 43.3° and 50.5°, which matched well with the diffraction peaks of (1 1 1) and (2 0 0) for the Cu metal (JCPDS 04-0836). Figure 2b showed Cu 2p XPS spectra of the copper coating. There were only two spin-orbit splitting components of 2p1/2 (952 eV) and 2p3/2 (932 eV) without the satellite peaks around 943 eV, further indicating the state of metallic copper.

The XRD pattern showed that a new diffraction peak appeared at 38.3° (Figure 2a), corresponding to the (1 1 1) crystal plane of silver (JCPDS 04-0783). As shown in the Cu 2p XPS spectra (Figure 2b), the two characteristic peaks of metallic copper at 932 eV (Cu 2p_3/2_) and 952 eV (Cu 2p_1/2_) were observed for the Cu-Ag chip like that in the case of the Cu chip. Due to the covering by the thin Ag layer, the intensities of these Cu peaks were decreased slightly. The XPS Ag 3d spectra (Figure 2c) of the Cu-Ag-coated fabric showed two peaks at 374 eV (Ag 3d_3/2_) and 368 eV (Ag 3d_5/2_), indicating the deposition of metallic silver.

Figure 2d showed the VIS-NIR diffuse reflectance spectra of the Cu-coated fabrics with different deposition times of Ag. The surface plasmon resonance (SPR) peak of Cu coating appeared at 556 nm, and the introduction of Ag enhanced this absorption and generated a new SPR peak in the near infrared region. Along with the growing of the Ag shell, the SPR peak in the near infrared region was blue shifted from 705 nm to 672 nm until it attenuated the SPR peak of Cu to merge into a single peak. This might influence the SERS performances of the chip, as discussed later.

### 3.2. SERS Performances of the Cu Chip

**Figure 3:**
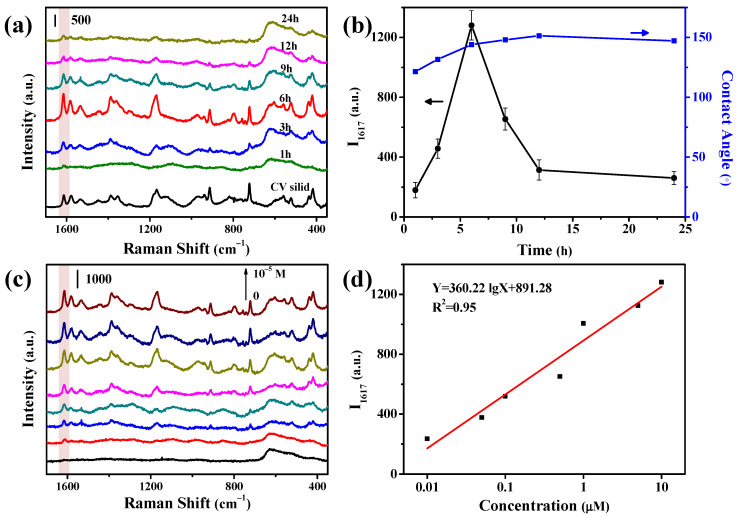
SERS performances of the Cu chip. (**a**) SERS spectra of 1 ppm CV on the Cu chips prepared with the different deposition time. (**b**) Influences of the Cu deposition time on the peak intensity of CV at 1617 cm^−1^ and the contact angle of the chip surface. (**c**) SERS spectra of CV at various concentrations on the Cu chips. (**d**) A plot of the peak intensity of CV at 1617 cm^−1^ against CV concentration.

In the Cu chip, metallic copper nanoparticles were deposited on the fabric and generated to a hydrophobic surface, which is possibly favorable to its SERS performance. As shown in Figure 3a, the Cu deposition time (and hence the deposition amount) influenced the SERS responses of CV on the Cu chip. By using the intensity of the strongest peak of CV at 1617 cm^−1^, the SERS signal intensity was plotted against the deposition time in Figure 3b. When increasing the deposition time from 0 to 6 h, the SERS signals of CV were rapidly increased from 0 to 1300 a.u.; further increasing the deposition time from 6 to 24 h decreased the SERS signal intensity, but finally keeping at 250 a.u. at about 24 h. The Cu deposition time mainly affected the individual Cu particles (the particle sizes, packing patterns) and the hydrophobicity of the surface. The hydrophobicity of the surface was monitored by measuring the contact angle. As shown in Figure 3b, once the Cu filmed was formed on the fabric, the contact angle was greater than 120°, showing hydrophobicity. For example, the contact angle of the chip surface with deposition time of 6 and 12 h was measured to be 144.0° and 150°, respectively. Because the deposition time dependence of the SERS signal intensity was greatly different from the deposition time dependence of the contact angle in the curve shape as shown in Figure 3b, the weak variation of the surface hydrophobicity would not be an important factor influencing the deposition time dependence of the SERS signal intensity. Therefore, the surface morphology of the chip was checked for different periods of deposition time. As shown in Figure 1a–c, the size of Cu NPs gradually increased and packed together with the prolonging of deposition time. When the deposition time is 1h (Figure 1a), the Cu NPs deposited on the fabric was small and loose with the size of 80 nm. When the deposition time was prolonged to 6 h (Figure 1b), the amount and diameter of Cu deposition on the fabric increased significantly, a large number of Cu NPs (100–200 nm) tightly packing to form hot spots. Further extended the deposition time to 12h (Figure 1c), the small size Cu NPs merged to form large Cu NPs with the size of around 300 nm, so the surface roughness and number of hot spots decreased. Based on the above discussions, the Cu deposition time was selected at 6 h hereafter. 

By using the sharp peak of CV at 1617 cm^−1^, the enhancement factor (EF) of the Cu chip was evaluated to be 2.0 × 106. Table 1 compared the EF values of the copper-based SERS chips prepared by various methods. This comparison indicated that the SERS performance of the presently developed Cu chip was somewhat poorer than that prepared by expensive physical methods (about 107), but much higher than that prepared by other chemical reduction methods (103–105). This was possibly related to the slow deposition of Cu in the present work, leading to more tightly packing of Cu NPs for the generation of more hot spots in the chip. In our method, no any organic stabilizing agents were used, and hence the Cu-coated fabric exhibited a clean background without interference peaks (Figure 3c). We measured the SERS spectra of CV at different concentrations (Figure 3c), and plotted the peak intensity at 1617 cm^−1^ against the CV concentration (Figure 3d). It was found that the SERS intensity was linearly correlated with the logarithm of CV concentration in the range of 10^−8^–10^−5^ M, with a LOD of 10^−8^ M.

### 3.3. SERS Performances of the Cu-Ag Chip

As shown in Table 1, the SERS performance of the presently prepared Cu chip needs to be promoted further in comparison with the best ones. More importantly, we found that the SERS performance of the Cu chip was decreased greatly after it was stored in air for several weeks due to its poor oxidation resistance (more details will be described in the next section). Therefore, it was required to further increase the SERS enhancing effect and the oxidation resistance of the SERS substrate. As we know, metallic Ag has stronger intrinsic SERS effect and is much more inert in air than metallic Cu. Therefore, our strategy was to cover a very thin layer of nano-Ag on the Cu chip by immersion plating.

**Figure 4:**
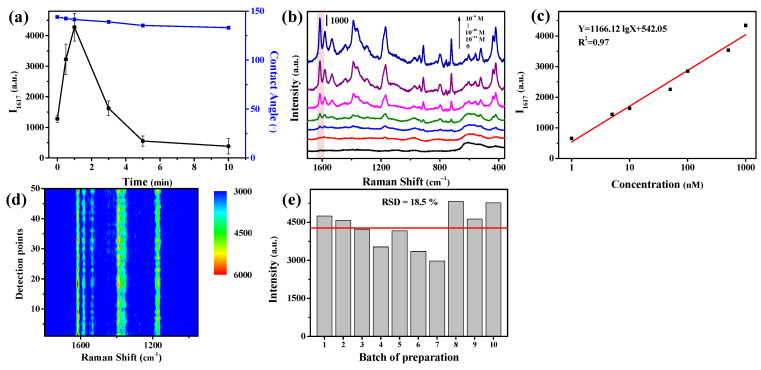
SERS performance of the Cu-Ag chip. (**a**) Effects of the replacement time of sliver on the peak intensity of CV at 1617 cm^−1^ and the contact angle of the chip surface. (**b**) SERS spectra of CV at various concentrations on the Cu-Ag chip. (**c**) A plot of peak intensity of CV at 1617 cm^−1^ on the Cu-Ag chip against CV concentration. Reproducibility of SERS signals obtained on the (**d**) intra-batch and (**e**) inter-batch Cu-Ag chips.

As shown in Figure 4a, as the replacement time of Ag was prolonged from 0 to 1 min, the Ag-modified Cu chip yielded a fast increasing of the SERS signal intensity (at 1617 cm^−1^) from 1281 to 4275 a.u. together with an increase of the enhancement factor to 7.6 × 106. This was explained by considering the effects of the deposited Ag: the deposited Ag NPs increased the surface roughness (as confirmed by the SEM observation in Figure 1e), being favorable to producing more hot spots; the initially deposited Ag NPs had a SPR peak at 705 nm, which matched better with the laser excitation of 785 nm. When the replacement time of Ag was more than 3 min, the SPR peak blue shifted, and its SERS performance decreased. This may because that the Ag covered the Cu, shielding the contribution of inner Cu. Figure 1e–g showed the SEM image of Cu-Ag chip with the replacement time of 1–5 min. With the prolonging of replacement time of Ag from 1 to 5 min, the Cu further dissolved, the Ag became larger to completely coat the Cu NPs (Figure 1f) and finally generated the Ag particles in micro scale (Figure 1g). Beyond that, Ag with higher surface energy was more easily wetted by water than Cu. Therefore, the contact angle of the Cu-based chip decreased to 141.5° with the increase of silver content, and finally tended to be stable due to the Ag completely covering the Cu (Figure 4a). Therefore, in the present work, the Ag deposition time was selected at 1 min for preparing the Cu-Ag chip. 

The SERS spectra of CV at various concentrations were recorded on the Cu-Ag chip as shown in Figure 4b. In comparison with the spectra recorded on the Cu chip for the specified individual concentrations of CV (Figure 3c), the spectra recorded on the Cu-Ag chip were much enhanced in the peak intensity. From the plot of the peak intensity at 1617 cm^−1^ against the CV concentration (Figure 4c), it was found that there was a linear correspondence between the SERS intensity and the logarithm of CV concentration in the range of 10^−9^–10^−6^ M, with an LOD as low as 10^−11^ M. 

In order to evaluate the uniformity of the as-prepared SERS chips, 50 points on the same Cu-Ag chip were selected randomly to detect the SERS signals. As shown in Figure 4c, the signal intensity of CV, especially for its strongest peak at 1617 cm^−1^, was very close to each other. Furthermore, we acquired the SERS spectra of CV at 1 mg·L^−1^ on ten Cu-Ag chips as shown in Figure 4d, and found that these spectra were also very close to each other with the relative standard deviation of only 12.6%. These demonstrated that the presently developed Cu-Ag chips have good reproducibility in term of both intra- and inter-batches.

### 3.4. Stability of the Cu-Based SERS Chips

**Figure 5:**
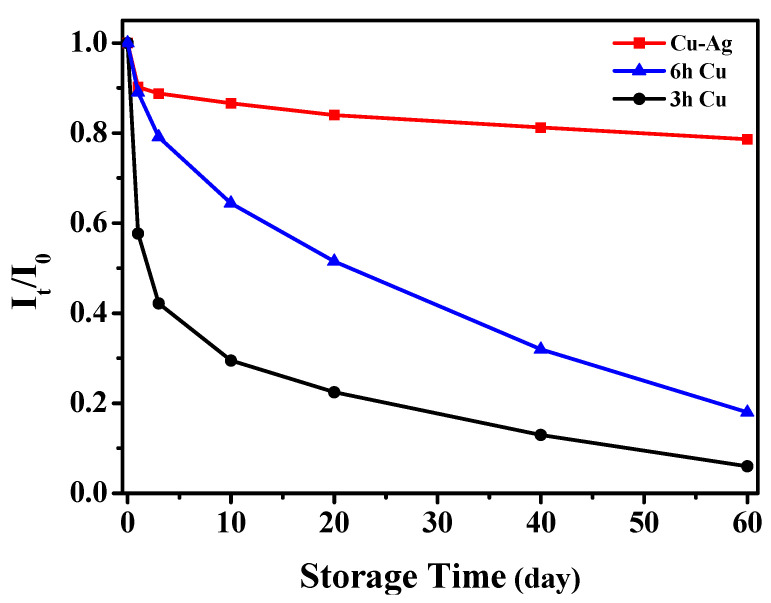
Endurances of SERS performances of various SERS chips to the air oxidation during storage: Cu(3 h) chip, Cu(6 h) chip and Cu-Ag chip.

The resistance of copper to air oxidation is not so good, and the oxidative corrosion of Cu may decrease the SERS performances of the Cu chips. By using the characteristic peak of CV at 1617 cm^−1^ as a reference, we recorded the original peak intensity on the newly prepared chip (I0) and the peak intensity (It) after the chip was stored in the air for a specified period time of t, and then used the ratio of It/I0 to evaluate the resistance of the chip to air oxidation. Here, three different chips were tested: the first chip was a Cu chip obtained with a Cu deposition time of 3 h, being referred to as Cu(3 h) chip, the surface of which had a contact angle of 131.7°; the second was a Cu chip obtained with a Cu deposition time of 6 h, being referred to as Cu(6 h) chip, the surface of which had a contact angle of 144.0°; the third was a Cu-Ag chip that was obtained by depositing Ag for 1 min on the Cu(6 h) chip, and it showed a contact angle of 141.0°. As shown in Figure 5, on the Cu(3 h) chip, the deposited Cu particles on the fabric were less densely packed and less hydrophobic, and the air oxidation during the storage of the chip was serious, which led to a fast decrease of the It/I0 ratio, only 6% of the response ability was kept after storing 60 days. In contrast, due to the much improved deposition of Cu, the decrease of the I_t_/I_0_ ratio on the Cu (6 h) chip became considerably slower. For the Cu-Ag SERS chip, its SERS performance maintained about 80% after storing in air for 60 days. It indicated that the Cu-Ag chip with good hydrophobicity exhibited high oxidation resistance.

**Figure 6:**
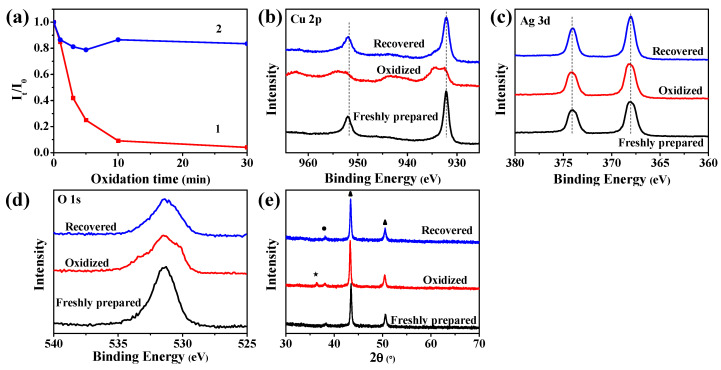
Recovering of the oxidized Cu-Ag SERS chip. (**a**) Dependence of the relative SERS intensity (I_t_/I_0_) of the Cu-Ag chip on oxidation time (curve 1) and the recovered value after a vacuum drying treatment of the oxidized chip (curve 2). (**b**–**d**) XPS spectra of the freshly prepared, oxidized and recovered Cu-Ag chips: (**b**) Cu 2p, (**c**) Ag 3d, and (**d**) O 1s envelops. (**e**) XRD patterns of the freshly prepared, oxidized and recovered Cu-Ag chips.

We also evaluated the stability of the SERS substrate by using an ethanol solution of H_2_O_2_ (0.01 M) to accelerate the oxidation of the substrate as shown in Figure 6a (curve 1). With the increase of oxidation time, the SERS signal intensity (in term of the I_t_/I_0_ ratio) of the as-prepared SERS chip (i.e., the oxidized chip) was rapidly decreased. When the oxidation time was 30 min, the relative intensity of CV was decreased to 4% of the original one. It was very interesting that the great loss of the oxidized chip in the SERS signaling could be substantially recovered by an after-treatment through vacuum drying as shown in Figure 6a (curve 2). For example, the vacuum drying treatment could recover the relative SERS intensity of the chip being oxidized for 30 min from 4% to about 79%. 

To understand what happened during the oxidation and the recovering treatment, we characterized the freshly prepared, oxidized and recovered Cu-Ag chips by using XPS and XRD (Figure 6b–e). As with that being discussed for Figure 2b, in the Cu 2p XPS spectrum of the freshly prepared Cu-Ag chip there were two characteristic peaks of metallic copper at 952 eV (Cu 2p_1/2_) and 932 eV (Cu 2p_3/2_), both of which being attributed to metallic Cu. However, in the spectrum of the oxidized chip (Figure 6b), the two peaks at 952 eV and 932 eV broadened towards the high binding energy region, indicating part of he copper converted to Cu(II), and two satellite peaks of Cu(II) appeared at 943 eV and 963 eV. These suggested that the oxidation induced the generation of Cu(II) as the oxidation product. The recovering treatment made the spectrum of the oxidized chip almost the same as that of the freshly prepared Cu-Ag chip. This meant that the recovering treatment by vacuum drying eliminated the oxidation product Cu(II) species.

Similarly, as discussed for the XPS Ag 3d spectrum of the fresh Cu-Ag chip in Figure 2c, the two peaks at 374 eV (Ag 3d_3/2_) and 368 eV (Ag 3d_5/2_) were attributed to the deposited metallic silver. The oxidation of the chip did not induce observable changes in the Ag 3d XPS spectrum, and the recovering treatment yielded yet no observable changes in the Ag 3d XPS spectrum (Figure 6c). These hinted that neither the oxidation-induced loss of the chip in the SERS sensing nor its recovering was directly related to the deposited Ag particles.

As shown in Figure 6d, in the XPS O 1s spectrum of the fresh Cu-Ag chip, a peak was observed at 532 eV, which was attributed to organic C-O bonding of fabric. After the chip was oxidized for 30 min, this peak became much broader, and its decovolution indicated that the oxygen was attached on the metal of Cu-Ag chip after oxidization, among these peaks, those at 534 eV and 530 eV were assigned to the adsorbed oxygen and metal oxides, respectively [36,37]. After the recovering treatment, both the peaks at 534 eV and 530 eV were much depressed in the intensity. This suggested that the oxygen attached on metal was partly removed.

The XRD patterns of the freshly prepared Cu-Ag chip are displayed in Figure 2a and were discussed before. After the oxidation, the oxidized Cu-Ag chip exhibited a new peak from cupric oxide as shown in Figure 6e. The new peak at 36.4° was assigned to the diffraction peak of (1 1 1) for the Cu_2+1_O (JCPDS 05-0667). Cu_2+1_O was Cu_2_O with metal excess defects, indicating that the O atom of it was easy to lose and lead to the oxygen vacancy [38,39]. A substantial part of the oxygen could be removed by the vacuum drying treatment. As confirmed by the XRD analysis, the diffraction peak of Cu_2+1_O for the oxidized chip was disappeared in the XRD patterns of the recovered Cu-Ag chip. These results showed that the O atom of surface with low binding energy could be removed by vacuum drying, which substantially recovered the SERS performance of the oxidized Cu-Ag SERS chip.

### 3.5. Quantitative SERS Detection of Other Organic Compounds with the Cu-Ag Chip

As discussed in Section 3.3 “SERS performances of the Cu-Ag chip”, a SERS method was developed by using the Cu-Ag chip for the detection of CV, which gave a linear correspondence between the SERS intensity and the logarithm of CV concentration in the range of 10^−9^–10^−6^ M, with a LOD as low as 10^−11^ M (Figure 4b,c). Here, we used this chip to detect more organic compounds to demonstrate its generality as a good SERS sensor. 

**Figure 7:**
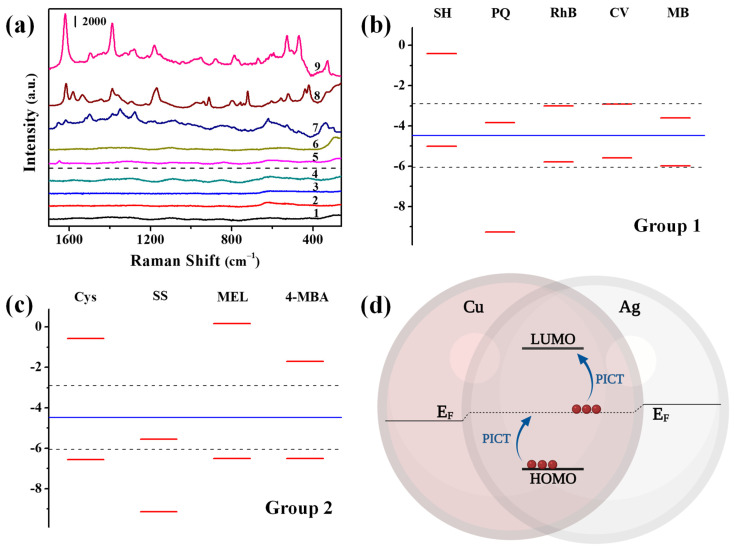
(**a**) SERE spectra of (1) Cys, (2) SS, (3) MEL, (4) 4-MBA, (5) SH, (6) PQ, (7) RhB (8) CV and (9) MB at 1 ppm on the Cu-Ag SERS chip. The energy level diagram of the molecules in (**b**) Group 1 and (**c**) Group 2. (**d**) The electron transition model between Cu-Ag chip and target molecules.

As shown in Figure 7a, the SERS spectra of nine organic compounds with relatively strong Raman activity were acquired at 1 ppm. It was found that five of them could be detected on the Cu-Ag chip, including sibutramine hydrochloride (SH), paraquat (PQ), rhodamine b (RhB), crystal violet (CV) and methylene blue (MB), which were classified as Group 1. In contrast, the other four (cysteine (Cys), saccharin sodium (SS), melamine (MEL) and 4-mercaptobenzoic acid (4-MBA)), being classified as Group 2, could not be detected on this chip. This difference indicated that the Cu-Ag chip had selective chemical enhancement for different targets due to CM. The CM of Raman scattering was ascribed to the chemical interaction between metal-molecule, whereby the charge transfer (CT) in the metal-molecule system was supposed to alter the electron density distribution of molecules, resulting in greater polarizability and thus enhanced Raman scattering. The energy levels of HOMO/LUMO of the above targets were calculated by Gaussian method, and it was found that the minimum energy barrier for the charge transfer between metal and molecules in Group 1 (Figure 7b) were in the range of 0.54–1.31 eV, being considerably smaller than those of the molecules in Group 2 in the range of 2.03–4.66 eV (Figure 7c). Therefore, a charge transfer is easily induced by irradiating 785 nm laser, leading to the photo-induced charge transfer (PICT). The charge transfer between the metal-molecule pair may occur in the direction of metal-to-molecule or molecule-to-metal, which depends on the Fermi level of metal and the HOMO/LUMO levels of the molecule (Figure 7d) [37,38,39]. In the Cu-Ag chip, the work function of Cu (4.65 eV) is higher than that of Ag (4.3 eV), and the electrons will transfer from Ag to Cu until the energy thermodynamic equilibrium level. Moreover, the Fermi level of Ag will be descended, and that of Cu will be raised up to attain the equilibrium level of −4.475 eV. To achieve PICT by the laser of 785 nm (ΔE = 1.58 eV), the target molecules should meet the requirement: HOMO level in the range of −4.475 eV to −6.055 eV, or LUMO level in the range of −2.895 eV to −4.475 eV. The molecules in Group 2 could not meet the above requirement, and hence there were no characteristic peaks in their SERS spectra obtained on the Cu-Ag chip even at a concentration of 100 ppm, while the molecules in Group 1 meet the requirement and could all be detected at 1 ppm. Moreover, the stronger charge transfer will be achieved as the energy level is closer to Fermi level of Cu-Ag chip. Due to the similar EM of substrate for adsorbed targets, the larger EF typically was achieved when the energy level of molecule was closer Fermi level. For example, the EF value of SH, MB, CV and RhB was 3.7 × 10^7^, 2.9 × 10^7^, 7.6 × 10^6^ and 1.2 × 10^6^ generally following an decreasing order in their minimum |ΔE| between the HOMO/LUMO of the molecules and Fermi of metal (0.54 eV, 0.87 eV, 1.10 eV and 1.31 eV). This clearly explained why the Cu-Ag chip had a selectively to the detection of different target molecules.

**Figure 8:**
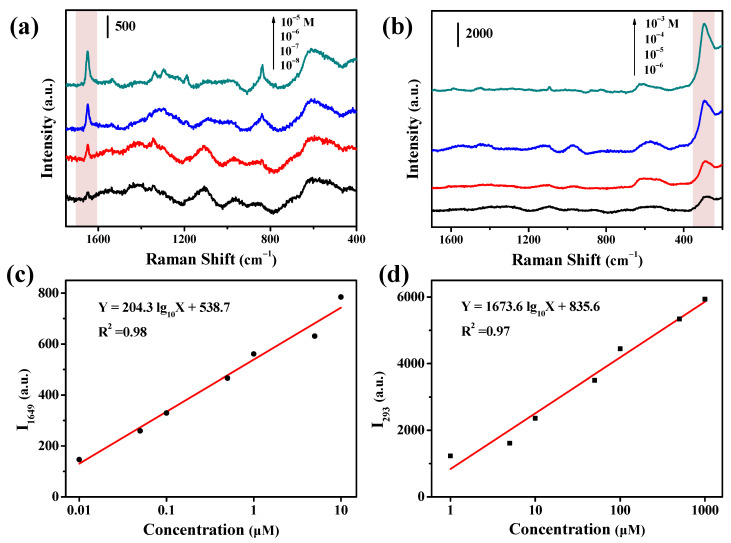
SERS spectra of (**a**) PQ and (**b**) SH at various concentrations on the Cu-Ag SERS chip. Linearity between the SERS intensity and log_10_ (concentration) of (**c**) PQ and (**d**) SH by using their strongest peaks.

In comparison with CV, the SERS activity of SH and PQ were much weaker, and hence we checked their quantitative detection to further evidence the merits of the Cu-Ag chip (Figure 8). As their concentration was increased, their characteristic peaks became stronger. By using the strongest peak of each of the tested compounds, the SERS intensity was plotted against the logarithm of its concentration. It was found that the SERS intensity was well linearly correlated with the concentration in log scale for each compound. The linear range and LOD values were evaluated to be 10^−8^–10^−5^ M and 10^−9^ M for PQ, 10^−6^–10^−3^ M and 10^−7^ M for SH, respectively. The wide linear ranges and low LOD values for all the tested targets indicated that the use of the Cu-Ag SERS chip provided a good quantitative detection method for SERS analysis.

## 4. Conclusions

In the present work, we have developed a sensitive and stable copper-based SERS chip by a chemical reduction method. The chemical deposition of Cu NPs on the fabric support ensured the Cu chip had no background interference of stabilizers because no stabilizers were used in the preparation. This permitted the quantitative detection of CV in a range from 10^−8^ M to 10^−5^ M with an LOD as low as 10^−8^ M. By considering the further improvement of the SERS enhancing effect and the air oxidation resistance of the Cu chip, a very thin layer of Ag NPs was deposited on the Cu chip by a replacement reaction. The introduction of Ag further enhanced the SERS performance of Cu chip, and the EF was increased from 2.0 × 10^6^ to 7.6 × 10^6^ after modification with Ag. Both the hydrophobicity and Ag shell improved the oxidation resistance of the Cu-Ag SERS chip, the SERS intensity kept 80% of the original signal after storing in the air for 60 days. The O atoms on the oxidized substrate could even be removed by vacuum drying to restore its SERS performance. The as-prepared Cu-Ag chip was used for detection of various targets, and results indicated that the molecules with required energy levels could be selectively detected at 1 ppm due to the CM caused by photo-induced charge transfer. The SERS method on this Cu-Ag chip also showed a wide linearity range and low LOD values for the quantitative detection of these organic compounds.

## Figures and Tables

**Table 1:** Comparison between copper-based SERS chips prepared by various methods.

SERS Substrate	Preparation Method	Targets	LOD (M)	EF	Ref.
Cu/gCN	pulsed laser ablation	CV R6G	10^−7^ 10^−6^	7.2 × 10^7^ 1.3 × 10^7^	[12]
Cu NPs/Si wafer	Si–H bond assembly	R6G	10^−9^	2.3 × 10^7^	[14]
Cu-doped glass	thermal annealing	RhB	10^−9^	1.5 × 10^8^	[15]
Cu NP arrays	ion-sputtering deposition	4-ATP	10^−7^	1.6 × 10^7^	[16]
Cu@G-NGNs	chemical vapor deposition	R6G	10^−7^	1.1 × 10^6^	[20]
Cu NPs	aerosol direct writing	RhB	10^−6^	2.1 × 10^5^	[28]
nanoporous Cu	subsequent dealloying	R6G	10^−9^	4.7 × 10^7^	[29]
Cu nanoislands	magnetron sputtering	4-ATP	10^−7^	4.0 × 10^4^	[30]
mesoporous Cu films	electrochemical deposition	R6G	10^−6^	3.8 × 10^5^	[27]
Cu/rGO	chemical reduction with rGO as stabilizing agent	CV	/	/	[17]
Cu NPs	chemical reduction with gelatin as stabilizing agent	CV	/	3.6 × 10^3^	[25]
Cu NPs	chemical reduction with octadecylamine as stabilizing agent	RhB	/	8.6 × 10^3^	[26]
3D nanoporous Cu leaves	chemical reduction with isooctane/PEG as stabilizing agent	4-MBA	/	1.2 × 10^6^	[11]
Cu-coated fabric Cu-Ag-coated fabric	chemical reduction without any stabilizing agent	CV	10^−8^ 10^−11^	2.0 × 10^6^ 7.6 × 10^6^	This work
